# Immunoactivating Peptide P4 Augments Alveolar Macrophage Phagocytosis in Two Diverse Human Populations

**DOI:** 10.1128/AAC.00742-13

**Published:** 2013-09

**Authors:** Mathieu Bangert, Adam K. Wright, Jamie Rylance, Matthew J. Kelly, Angela D. Wright, George M. Carlone, Jacquelyn S. Sampson, Gowrisankar Rajam, Edwin W. Ades, Aras Kadioglu, Stephen B. Gordon

**Affiliations:** Respiratory Infection Group, Liverpool School of Tropical Medicine, Liverpool, United Kingdoma; Malawi-Liverpool-Wellcome Clinical Research Laboratories, Blantyre, Malawib; Division of Bacterial Diseases, Centers for Disease Control and Prevention, Atlanta, Georgia, USAc; Clinical Infection, Microbiology and Immunology, Institute of Infection & Global Health, University of Liverpool, Liverpool, United Kingdomd

## Abstract

New treatment strategies are urgently needed to overcome early mortality in acute bacterial infections. Previous studies have shown that administration of a novel immunoactivating peptide (P4) alongside passive immunotherapy prevents the onset of septicemia and rescues mice from lethal invasive disease models of pneumococcal pneumonia and sepsis. In this study, using two diverse populations of adult volunteers, we determined whether P4 treatment of human alveolar macrophages would upregulate phagocytic killing of Streptococcus pneumoniae *ex vivo*. We also measured macrophage intracellular oxidation, cytokine secretion, and surface marker expression following stimulation. Peptide treatment showed enhanced bacterial killing in the absence of nonspecific inflammation, consistent with therapeutic potential. This is the first demonstration of P4 efficacy on *ex vivo*-derived human lung cells.

## TEXT

The rate of early mortality from severe pneumonia and sepsis is high in developed and developing countries. Antibiotic treatment alone is inadequate for severe infections, as treatment has not been shown to reduce deaths that occur in the initial 48-hour period following admission (1). To bridge this treatment window, augmented passive immunotherapy (API) is a potential new treatment paradigm that uses the concurrent administration of an immunoactivating peptide (P4) to enhance phagocytosis with pathogen-specific antibodies in pooled immunoglobulin (IVIG). P4 is a 28-amino-acid (aa) peptide derived from the functional site of the Streptococcus pneumoniae pneumococcal surface adhesion A protein (PsaA) (2). Murine models of acute infection have demonstrated the therapeutic benefits of API administered by either intranasal or intravenous routes. Such treatment has been demonstrated to rescue mice from invasive pneumonia (3, 4), influenza-pneumonia coinfections (5), and methicillin-resistant Staphylococcus aureus (MRSA) (6), independent of age (7) and strain background (8), although to date there are no data from human lung samples. Since P4 peptide treatment of Streptococcus pneumoniae (pneumococcus)-infected murine lungs prevented the onset of sepsis and subsequent host mortality (4), we hypothesized that *ex vivo* P4 treatment of human alveolar macrophages would lead to enhanced phagocytic function with no or limited generalized inflammation.

**Fig 1:**
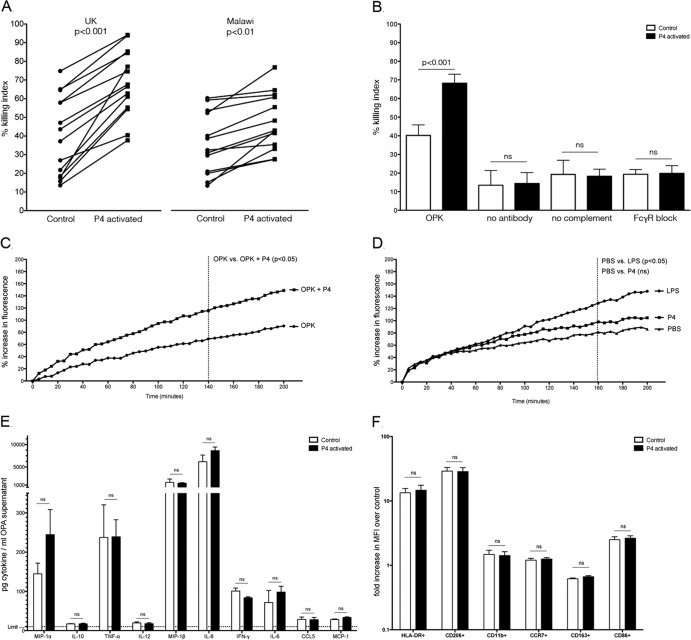
(A) Opsonophagocytosis killing assay (OPKA) using human alveolar macrophages from healthy volunteers recruited in Liverpool, United Kingdom (*n* = 14), and Blantyre, Malawi (*n* = 13); (B) OPKA of United Kingdom volunteers (*n* = 6), where “OPK” designates assays in which all components are included (macrophage, bacteria, antibody, complement), “no antibody” designates assays without the presence of antibody, “no complement” designates assays without the presence of complement, and “FcγR block” designates assays where Fcγ receptors were occupied by IgG prior to the assay; (C) intracellular oxidation of alveolar macrophages (*n* = 6; United Kingdom population) during an opsonophagocytosis assay in the presence (OPKA+P4) or absence (OPK) of peptide stimulation; (D) intracellular oxidation in the presence of PBS, LPS (100 μg/ml), or P4 (1 mg/ml) alone; (E) cytokine levels detected in OPKA supernatant using a BD Flex Set kit following a 2-h OPKA; (F) surface markers of activation on control or P4-activated alveolar macrophages (*n* = 6) following a 2-h OPKA, stained and analyzed using flow cytometry.

To test this hypothesis, healthy adult volunteers were recruited to a study in Liverpool (United Kingdom) and Blantyre (Malawi) involving bronchoscopy and bronchoalveolar lavage (BAL). Ethical approval was obtained from the National Health Service Research Ethics Committee (11/NW/0011) and the Malawi College of Medicine Ethics committee (P.03/11/1063). The median age of United Kingdom volunteers was 25.6 years ± 9.5 (8 females, 6 males) and that of Malawian volunteers was 31.4 years ± 3.3 (11 females, 2 males). Alveolar macrophages were isolated from BAL fluid collected during bronchoscopy by adherence and washing steps, resulting in >95% alveolar macrophage purity. An opsonophagocytosis killing assay (OPKA) was then performed as previously described (3) with minor modifications. Briefly, 1 × 10^5^ macrophages were allowed to phagocytose IVIG-opsonized Streptococcus pneumoniae strain D39-ST2 (IVIG final dilution, 1:32; multiplicity of infection, 1:100) in the presence of complement for 2 h. P4 peptide solution (10 μl of 1 mg/ml solution) was added to the OPKA mixture following the preopsonization stage, and CFU were cultured from supernatants following a 2-h incubation. A bacterial killing index was established by comparing the number of pneumococci recovered after OPKA to the number of pneumococci added. In some reactions, Fcγ receptors were occupied using human IgG prior to OPKAs to assess the involvement of Fcγ receptors during P4-augmented phagocytosis. In line with previous data on murine macrophages (4), peptide stimulation of human alveolar macrophages led to significantly enhanced bacterial killing in both the United Kingdom (40.1% killing for control versus 68.2% killing for P4 treated; *P* < 0.001, analysis of variance [ANOVA]; *n* = 14 volunteers) and Malawian (35.8% killing for control versus 47.6% for P4 treated; *P* < 0.01, ANOVA; *n* = 13 volunteers) populations (Fig. 1A). In contrast, phagocytosis in the absence of antibody or complement was reduced to 10.8% or 20.1%, respectively, and no detectable differences between stimulated and nonstimulated groups were seen (*n* = 6; United Kingdom population). Similarly, macrophages in which Fcγ receptors were occupied following stimulation showed reduced phagocytosis (23.3%), and no detectable differences between stimulated and nonstimulated groups were seen (Fig. 1B). There is therefore a necessity for all components involved in phagocytosis (immunoglobulin, complement, phagocytic receptors) to be present for P4 treatment to be effective.

Alveolar macrophages respond to stimuli and infection with increased oxidative metabolism. We assessed whether peptide treatment would alter intracellular oxidation in the presence and absence of immune complexes using a technique previously described (9). Briefly, alveolar macrophages were first incubated with 2′,7′-dichlorofluorescein-diacetate (DCFH-DA), followed by lipopolysaccharide (LPS) (100 μg/ml solution; Sigma, United Kingdom) or P4 solution (10 μl of 1 mg/ml solution). Intracellular oxidation was then measured using a microplate reader (BMG Labtech, United Kingdom). The assay was also performed with opsonized pneumococci to measure intracellular oxidation in response to P4-mediated phagocytosis. P4 treatment of alveolar macrophages exposed to opsonized bacteria and complement (*n* = 6; United Kingdom population) led to a significant increase in fluorescence (oxidation) compared to that of the control (135% for the treated versus 81% for the control at 140 min; *P* < 0.05, ANOVA; *n* = 6) (Fig. 1C). Direct stimulation of alveolar macrophages by P4 peptide in the absence of an immune complex, however, showed no statistical difference (102% for treated versus 84% for control at 160 min; *P* = 0.30, ANOVA) (Fig. 1D). Direct stimulation with LPS alone, in comparison, significantly increased intracellular macrophage oxidation compared to that of nontreated control cells (139% for LPS treated versus 84% for control at 160 min; *P* < 0.05, ANOVA). In addition, using detergent to burst open cells following OPKA, we found <2% of the inoculum intracellularly, with no significant difference between stimulated and unstimulated control macrophages (data not shown). These data together suggest that peptide stimulation also leads to effective breakdown of internalized pneumococci.

Since peptide stimulation of alveolar macrophages enhanced bacterial killing and intracellular oxidation, we investigated whether peptide treatment would alter expression of surface markers and the release of cytokines during an OPKA. Following OPKA, alveolar macrophages were stained using optimal concentrations of anti-HLA-DR antibody (major histocompatibility complex class II [MHC-II]; 560743), CD206 (mannose receptor; 55089), CD11b (integrin; 347557), CCR7 (chemokine receptor; 557648), CD163 (activation marker; 556018), and CD86 (activation marker; 555657), and supernatants were analyzed using the BD cytokine bead array kits for soluble proteins (BD-CBA Flex Sets) according to the manufacturer's instructions. Following a 2-h incubation with opsonized pneumococci and complement, alveolar macrophages (*n* = 11; United Kingdom population) did not significantly alter their secretion of inflammatory cytokines, MIP-1β, monocyte chemoattractant protein 1 (MCP-1), interleukin 10 (IL-10), IL-12, gamma interferon (IFN-γ), tumor necrosis factor alpha (TNF-α), or CCL5 when stimulated with P4 (*P* > 0.1). A slight increase of IL-8 (*P* = 0.08), MIP-1α (*P* = 0.1), and IL-6 (*P* = 0.1) was detected following peptide stimulation, but this was not statistically significant. Similarly, following a 2-h incubation in an OPKA, P4-treated alveolar macrophages (*n* = 6; United Kingdom population) did not express higher levels of HLA-DR, CD206, CD11b, CCR7, CD163, or CD86 than control cells (*P* > 0.1). These data suggest that P4-enhanced phagocytic killing does not induce changes in the activation profile of alveolar macrophages (Fig. 1E and F).

Previous work has shown that targeting effector cells during pneumonia via direct peptide administration to affected lungs enhances the clearance of infection and prevents the onset of septicemia (4). During pneumonia, neutrophils and alveolar macrophages are the predominant responding phagocytes. We have previously shown that human peripheral blood neutrophil phagocytosis is augmented following peptide stimulation (3), and here we present the first data showing human alveolar macrophages, critical to lung defense, responding to peptide stimulation. In this study, alveolar macrophages from both United Kingdom and Malawian volunteers showed a significant increase in bacterial killing and intracellular oxidation as a result of peptide treatment. Two diverse human populations with different levels of susceptibility to pneumococcal disease were deliberately compared. United Kingdom adults of the age recruited have a low incidence of pneumonia (<1/1,000/year) and pneumococcal carriage (<10%), but in Malawi, disease incidence is significantly higher, due to greater exposure and risk factors, including malnutrition, smoke exposure, and concurrent infections (10).

In summary, we have shown that P4 peptide treatment of human alveolar macrophages led to a significant increase in their ability to phagocytose opsonized pneumococci. Importantly, we show P4 efficacy in two diverse human populations with differing levels of susceptibility to pneumococcal disease. These data are consistent with rescue from pneumonia and sepsis, as demonstrated in murine models. Our findings are important, as early treatment options for severe sepsis are urgently needed, and API offers a new paradigm for treatment of invasive diseases such as sepsis. As API is an antibody-driven treatment, in the presence of effective opsonization, peptide stimulation will lead to enhanced phagocytosis irrespective of the pathogen. Opsonization occurs through the broad specificity of IVIG, and the formed immune complexes are effectively cleared through the effects of peptide stimulation (including modulation of phagocytic Fcγ receptors) on phagocytes (4, 5). This has the potential to rapidly assist an infected patient prior to diagnostic results and antibiotic efficacy. We envisage P4 and immunoglobulin administration as part of an adjunct therapy with antibiotic treatment, a strategy that has previously been shown to rescue mice with lethal invasive pneumonia (8).

To our knowledge, this is the first demonstration of exogenous peptide treatment leading to increased opsonophagocytosis of bacteria by human alveolar macrophages. Together with our previous studies, we have now shown that P4 therapy is highly effective in stimulating major phagocytes involved in defending against pneumonia and sepsis in both *in vitro* human and *in vivo* murine studies. Augmented passive immunotherapy is a promising new strategy for clinical testing within a broad range of patient groups suffering from respiratory infections.
